# Roles of Water Molecules in the Structures and Magnetic Properties of Coordination Polymers with a Dicarboxylate Ligand

**DOI:** 10.3390/ma18051089

**Published:** 2025-02-28

**Authors:** Dehui Zong, En-Qing Gao, Dawei Zhang

**Affiliations:** State Key Laboratory of Petroleum Molecular & Process Engineering, Shanghai Key Laboratory of Green Chemistry and Chemical Processes, School of Chemistry and Molecular Engineering, East China Normal University, Shanghai 200062, China; 51254300011@stu.ecnu.edu.cn

**Keywords:** coordination polymers, water bridges, hydrogen bonds, magnetic properties, nickel, cobalt, spin canting

## Abstract

Three new coordination polymers, {[M(nbpdc)(DMF)(H_2_O)_2_]·H_2_O}_∞_ (M = Co and Ni) and [Zn(nbpdc)(DMF)(H_2_O)]_∞_, were synthesized from 2-nitrobiphenyl-4,4′-dicarboxylate (nbpdc^2−^). The isomorphous Co(II) and Ni(II) compounds exhibited a two-dimensional coordination network in which the chains with single-water bridges and the chains with single-nbpdc^2−^ bridges intersected each other by sharing the metal ions. The coordination networks were connected with uncoordinated water molecules through hydrogen bonds. The rarely identified single-water-bridged coordination chain was reinforced by water-based intrachain hydrogen bonds. The single-water bridges mediated modest antiferromagnetic superexchange in both Co(II) and Ni(II) compounds and afforded a spin-canting structure for the Co(II) compound at low temperatures. Water molecules played a distinct structural role in the Zn(II) compound, which was a one-dimensional coordination polymer with single-nbpdc^2−^ bridges. Instead of bridging metal ions, each water molecule was coordinated to one metal ion and hydrogen-bonded to the coordination spheres of other two metal ions, resulting to an infinite ladderlike hydrogen-bonding motif. The ladders interlinked the nbpdc-bridged chains into a three-dimensional supramolecular architecture featuring the 5-conneted {4^4^.6^4^} net.

## 1. Introduction

Coordination polymers are currently attracting considerable attention, mainly because they can show intriguing adsorptive [1,2], catalytic [3,4], conductive [5,6], luminescent [7,8], and magnetic [9,10] properties and have great potential in various fields of technological applications [11]. In the context of molecular magnetism, coordination polymers provide versatile platforms for enhancing or reducing short-range and long-range magnetic correlations and coupling magnetism with other functions. A common and primary issue for the design of molecular magnetic materials, with a few exceptions such as single-ion magnets [12] and spin-crossover compounds [13,14], is the choice of effective bridges that can transmit magnetic exchange between paramagnetic centers. Various bridges, mainly monoatomic like oxo and hydroxo, diatomic like cyano, and triatomic like azido and carboxylate bridges, have been extensively studied for magnetic exchange between paramagnetic metal ions [15,16,17,18].

When coordinated to metal ions, the water molecule usually serves as a terminal ligand, but it can also bind two metal ions. The water coordination bridge is often observed to be coexistent with phenoxo [19,20,21,22,23,24], carboxylate [25,26,27,28,29,30,31,32], and diaza bridges [33,34,35]. The exclusively water-bridged motifs are still rare. The triple-water bridge has been found between diamagnetic metal ions in hexanuclear M_2_Cr_4_ (M = Sr(II), Pb(II)) clusters [36]. Double-water bridges have been observed in multinuclear complexes and coordination polymers [37,38,39,40], transmitting antiferromagnetic or ferromagnetic interactions. The single-water-bridged binuclear motifs and infinite chains have been identified in discrete complexes [41] and coordination polymers [42,43,44,45,46]. The single bridge propagates antiferromagnetic exchange for Mn(II), Co(II), and Ni(II) ions [43] while ferromagnetic exchange was reported for a Cu(II) chain [45] and a heterometallic CuNi chain [46]. Considering the ubiquity of water in biological and environmental processes, the exploration of new water bridging structures is always interesting, but it remains challenging because the molecule tends to deprotonate into hydroxo anions, which are more prone to bridging coordination.

In this context, we report here three coordination polymers derived from 2-nitrobiphenyl-4,4′-dicarboxylic acid (H_2_nbpdc) [47], focusing on the structural and magnetic roles of water. [Zn(nbpdc)(DMF)(H_2_O)]_∞_ (**1**) is a one-dimensional (1D) coordination polymer with single-nbpdc^2−^ bridges. The water molecule in it is terminally coordinated to one Zn(II) and hydrogen-bonded to another two Zn(II) spheres, affording unique 1D ladderlike motifs that arrange the nbpdc-bridged coordination chains into a three-dimensional (3D) five-connected supramolecular net. {[M(nbpdc)(DMF)(H_2_O)_2_]·H_2_O}_∞_ (M = Co (**2**) and Ni (**3**)) are two-dimensional (2D) coordination polymers. The coordinated water molecules serve as single bridges to build infinite M((H_2_O)]_∞_ chains that share metal ions with the nbpdc-bridged chains. Magnetic studies have suggested that the single-water bridge mediates modest antiferromagnetic exchange along the Co(II) and Ni(II) chains and leads to a systematically canted spin structure at low temperatures for the strongly anisotropic Co(II) chain.

## 2. Experimental Procedure

### 2.1. Materials and Methods

The ligand, 2-nitrobiphenyl-4,4′-dicarboxylic acid (H₂nbpdc), was synthesized following the literature method [48]. Other reagents used for synthesis and analysis were purchased from commercial sources (Macklin, Shanghai, China) and used without further purification. Elemental analyses were performed using a Vario ELIII Elemental Analyzer (Elementar, Frankfurt, Germany). FT-IR spectra were recorded in the range of 500–4000 cm⁻¹ using KBr pellets on a NEXUS 670 spectrophotometer (Nicolet, Madison, WI, USA). Magnetic measurements were conducted with a SQUID MPMS-5 magnetometer (Quantum Design, San Diego, CA USA) and diamagnetic corrections were applied using Pascal’s constants. Powder X-ray diffraction (PXRD) was measured on a D/Max-2500 diffractometer (Rigaku, Tokyo, Japan) with Cu Kα radiation.

### 2.2. Synthesis

- **[Zn(nbpdc)(DMF)(H_2_O)]_∞_ (1):** A suspension of H_2_nbpdc (0.050 mmol, 0.0145 g) and Zn(NO_3_)_2_·6H_2_O (0.20 mmol, 0.059 g) in DMF/H_2_O (1:1 (*v*/*v*), 2 mL) was stirred thoroughly for 30 min. The mixture was transferred to a 20 mL stainless steel reactor lined with Teflon and placed in an 80 °C oven for 3 days. After slow cooling to room temperature, pale yellow flake crystals of **1** were obtained in a yield of 87% based on Zn(NO_3_)_2_·6H_2_O. Elemental analysis calculated for C_17_H_16_ZnN_2_O_8_ (%): C 46.36, H 3.66, and N 6.36. Found(%): C 46.39, H 3.83, N 6.14. IR (KBr, cm^−1^): 3068 (m), 1653 (s), 1611 (s), 1532 (vs), 1488 (m), 1387 (s), 1351 (s), 1250 (w), 1185 (w), 1112 (m), 1005 (w), 919 (w), 862 (m), and 785 (m);
- **{[Co(nbpdc)(DMF)(H_2_O)_2_]·H_2_O}_∞_ (2):** The compound was obtained in the form of pink flake crystals by the procedure described above for **1,** except that Co(NO_3_)_2_·6H_2_O (0.20 mmol, 0.058 g) was used in the place of Zn(NO_3_)_2_·6H_2_O. Yield: 86% based on Co(NO_3_)_2_·6H_2_O. Elemental analysis calculated for C_17_H_20_CoN_2_O_10_ (%): C 43.33, H 4.28, and N 5.94. Found (%): C 43.19, H 3.79, N 5.63. IR (KBr, cm^−1^): 3435 (m), 1667 (s), 1581 (s), 1530 (vs), 1393 (s), 1351 (s), 1249 (w), 1179 (w), 1108 (w), 1003 (w), 787 (m), and 690 (m);
- **{[Co(nbpdc)(DMF)(H_2_O)_2_]·H_2_O}_∞_ (3):** The compound was obtained in the form of pale green microcrystals by the procedure described above for **1,** except that Ni(NO_3_)_2_·6H_2_O (0.20 mmol, 0.058 g) was used in the place of Zn(NO_3_)_2_·6H_2_O. Yield: 72% based on Ni(NO_3_)_2_·6H_2_O. Elemental analysis calculated for C_17_H_20_NiN_2_O_10_ (%): C 43.35, H 4.28, and N 5.95. Found(%): C 43.50, H 3.99, N 5.89. IR (KBr, cm^−1^): 3437 (m), 2973 (w), 1663 (s), 1580 (s), 1530 (vs), 1397 (s), 1351 (s), 1248 (w), 1176 (w), 1110 (w), 1059 (w), 1004 (w), 808 (m), 786 (m), and 692 (m).

### 2.3. X-Ray Crystallography

X-ray single-crystal diffraction data for complexes **1** and **2** were collected using a Bruker Apex II CCD area detector equipped with graphite-monochromated Mo Kα radiation (λ = 0.71073 Å). Empirical absorption corrections were applied using the SADABS program [49]. The structures were solved by direct methods and refined by the full-matrix least-squares method on *F*^2^ [50]. All non-hydrogen atoms were refined with anisotropic thermal parameters. Hydrogen atoms attached to carbon atoms were placed in calculated positions and refined using the riding model. The nitro group of the nbpdc^2−^ ligand in **2** was modeled with disorder over two benzene rings. A summary of the crystallographic data and refinement parameters is provided in Table 1. Structural visualization and geometrical analysis of interactions were performed using the *Diamond* program [51]. Topological analysis was performed using the *ToposPro* program [52].

## 3. Results and Discussion

### 3.1. Synthesis and Structural Characterization

**Figure 1:**
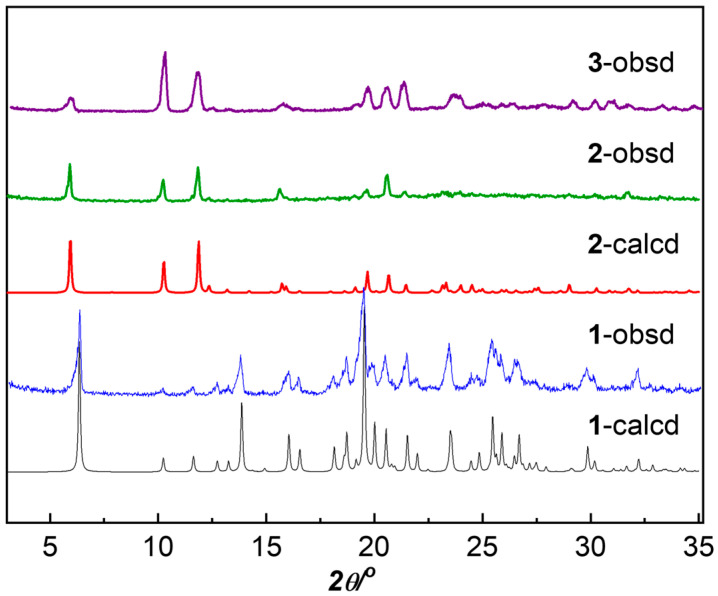
PXRD profiles of **1**–**3** compared with those simulated from the crystal data of **1** and **2**.

Compounds **1**–**3** were synthesized from H_2_nbpdc and zinc(II), cobalt(II), and nickel(II) nitrates, respectively, under the same solvothermal conditions in DMF and water. The powder X-ray diffraction (PXRD) patterns of **1** and **2** were different and in good agreement with those simulated from the single-crystal X-ray crystallographic data (Figure 1), supporting the phase purity of the bulk products. Single crystals of **3** suitable for structural determination were not obtained, but the similar PXRD patterns of **2** and **3** suggested that they were isomorphous. Consistently, the profiles of the IR spectra of **2** and **3** were very similar while that of **1** was obviously different (Figure S1). Nevertheless, they all showed the characteristic absorption bands for the DMF molecule (ν(C=O), 1653–1663 cm^−1^), the carboxylate group (ν_as_, 1611–1580 cm^−1^; ν_s_, 1387–1397 cm^−1^), and the nitro group (ν_as_, 1530 cm^−1^; ν_s_, 1350 cm^−1^).

**Figure 2:**
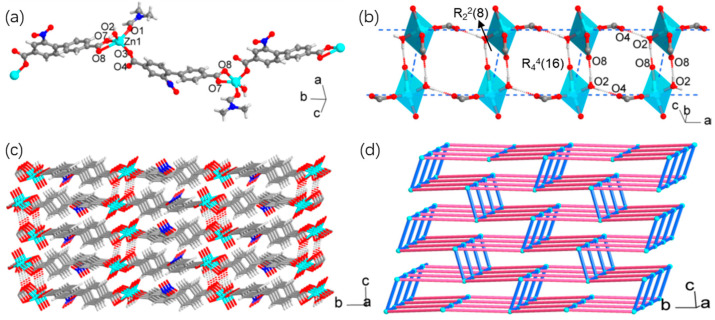
Crystal structure of **1**. (**a**) A view of the coordination chain formed from Zn(II) and nbpdc^2−^ (ellipsoidal probability: 50%). The hydrogen atoms of nbpdc^2−^ and DMF have been omitted for clarity. (**b**) The hydrogen-bonded ladderlike motif. (**c**) A view showing the stacking of the coordination chains through hydrogen bonds. The non-oxygen atoms of DMF and the nitro groups and hydrogen atoms of nbpdc^2−^ have been omitted for clarity. (**d**) A topologic representation of the 3D structure composed of coordination chains (red) and hydrogen-bonded ladders (blue).

According to X-ray crystallographic analysis, compound **1** exhibited an interesting 3D supramolecular architecture featuring 1D coordination chains and hydrogen-bonded ladders (Figure 2). The Zn(II) ion adopted a trigonal bipyramidal geometry completed by a chelating carboxylate, a monodentate carboxylate, a water molecule, and a DMF molecule. The axial positions were occupied by the oxygen of DMF and an oxygen atom of the chelating carboxylate. The distortion of the geometry mainly arose from the small bite angle (O7-Zn1-O8, 55.87(7)°) and the long axial bond distance (Zn1-O8, 2.572(2) Å) of the chelating carboxylate. Alternatively, the Zn(II) geometry could also be described as tetrahedral, excluding the weak Zn1-O8 interaction. The four short bond distances (1.918(2)–1.989(2) Å) were typical of the tetrahedral coordination of Zn(II). The nbpdc^2−^ ligand connected two Zn(II) ions through the monodentate and chelating carboxylate groups, giving rise to a 1D zigzag chain along the *b*-axis (Figure 2a).

The chains were linked into a 3D architecture through strong O-H···O hydrogen bonds between coordinated water and carboxylates (Table S1). Interestingly, the hydrogen bonds interconnected the [Zn(COO)_2_(H_2_O)(DMF)] coordination spheres into an infinite ladderlike motif extending along the *a-*axis (Figure 2b). The side rails of the ladder were constructed by the single hydrogen bonding between water and the uncoordinated oxygen of the monodentate carboxylate (H···O4, 1.82(5) Å) while the rung was formed by the complementary double hydrogen bonding between two centrosymmetrically related Zn(II) spheres, for which the weakly coordinated oxygens of the chelating carboxylates served as hydrogen acceptors (H···O8, 1.91(4) Å). The ladder consisted of two alternate hydrogen-bonding cycles, an eight-membered cycle with two hydrogen bonds (graph set R_2_^2^(8)), and a sixteen-membered cycle with four hydrogen bonds (R_4_^4^(16)). The hydrogen-bonded ladders and the nbpdc-bridged chains shared the Zn(II) spheres to build a 3D network (Figure 2c). In particular, the H···O4 hydrogen bonds along the ladder rails connected the chains into layers along the *ab*-plane, and the double H···O8 hydrogen bonds for the ladder rungs served to connect a layer with the upper and lower layers. Topological analysis was performed by taking the [Zn(COO)_2_(H_2_O)(DMF)] sphere as a node with five connections: three connections through hydrogen bonds and two connections through the biphenyl moieties. The analysis reduced the intricate supramolecular structure into a uninodal net with point symbol {4^6^.6^4^}, which clearly showcased how the 3D network is constructed from the nbpdc-bridged chains with the hydrogen-bonded ladders (Figure 2d).

**Figure 3:**
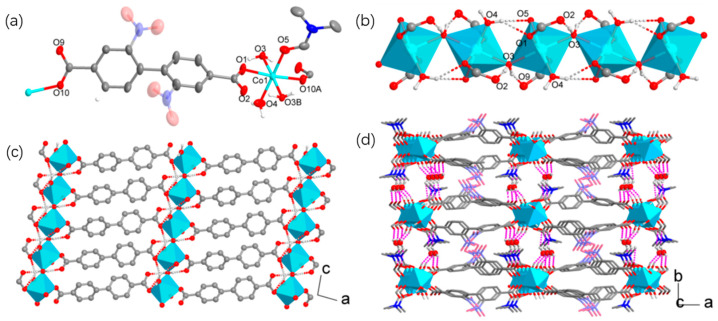
Crystal structure of **2**. (**a**) A view of the coordination modes of Co(II) and nbpdc^2−^ (ellipsoidal probability: 50%). The nitro group disordered over two positions is shown in light color and the hydrogen atoms of nbpdc^2−^ and DMF have been omitted for clarity. (**b**) A water-bridged chain assisted with hydrogen bonds. (**c**) A 2D coordination layer. The non-oxygen atoms of DMF and the nitro groups and hydrogen atoms of nbpdc^2−^ have been omitted for clarity. (**d**) A view of the layer packing showing interlayer hydrogen bonding. The hydrogen atoms of nbpdc^2−^ and DMF have been omitted for clarity.

By contrast, **2** exhibited a 2D coordination network. As shown in Figure 3a, the unique Co(II) ion was octahedrally coordinated with two *trans*-positioned monodentate carboxylate groups, two equivalent and *trans*-positioned water molecules (O3 and O3B), another water molecule, and a DMF molecule. The Co-O bonds for the O3 water molecules (2.157(3) and 2.182(3) Å) were obviously longer than other coordination bonds (2.041(3)–2.086(4) Å), defining an axially elongated Jahn–Teller distortion in the octahedral geometry.

The O3 water molecules served as single bridges to connect Co(II) into zigzag [Co(H_2_O)]_∞_ chains propagating along the *c*-axis (Figure 3b). The Co-O-Co bridging angle was 131.8(2)° and the Co···Co distance was 3.960(1) Å. The single-water bridging motif was reinforced by O-H···O hydrogen bonds (Table S1). The water bridge formed two strong hydrogen bonds (H···O, 1.85(2) and 1.74(2) Å) with the uncoordinated oxygen atoms of the carboxylate groups from adjacent Co(II) ions. In addition, the terminal water ligand (O4) bonded to one Co(II) used a hydrogen atom to form bifurcate hydrogen bonds (H···O, 2.23(9) and 2.43(2) Å) with two oxygen atoms from the carboxylate and DMF bonded to another Co(II). These hydrogen bonds should have provided additional stabilization energy for the formation of the single-water bridging structure.

The nbpdc^2−^ ligands connected Co(II) ions through monodentate carboxylate groups to generate metal–organic chains along the [201] direction. The Co···Co distance spanned by nbpdc^2−^ was 15.058(2) Å. The nbpdc-bridged and water-bridged chains intersected each other by sharing the metal ions, giving rise to 2D layers along the *ac*-plane (Figure 3c). The layers were packed in the ABAB fashion down the *b*-axis (Figure 3d), with the shortest interlayer Co···Co distance being 8.756(1) Å. The interlayer space was primarily occupied by the coordinated DMF molecules and the nitro groups of the organic ligands. The uncoordinated water was located at the interlayer aperture left and formed three hydrogen bonds (H···O, 1.94(3)–2.11(2) Å) with the water bridge and uncoordinated carboxylate oxygen atoms from adjacent layers. Accordingly, the uncoordinated water molecules could be regarded as hydrogen-bonding bridges connecting the coordination layers into a 3D architecture.

As can be seen, **1** was distinctly different from **2** and **3** in coordination and the hydrogen network, though they had been synthesized under the same conditions and all contained nbpdc-bridged coordination chains. The differences could be traced to the different coordination habits of the metal ions. The coordination geometry was primarily determined by the balance between various stabilization and destabilization effects such as attractive metal–ligand bonds and repulsive interligand interactions. According to the crystal-field theory, Co(II) (*d*^7^) and Ni(II) (*d*^8^) exhibit a higher crystal-field stabilization energy (CFSE) in an octahedral field than in a tetrahedral field whereas Zn(II) (*d*^10^) shows no CFSE in any field. The additional CFSE was conducive to the octahedral coordination of Co(II) and Ni(II), as observed in **2** and **3**. Without the CFSE to stabilize the octahedral coordination, Zn(II) could adopt the tetrahedral arrangement that exhibited stronger metal–ligand bonds and weaker interligand repulsion. In **1**, the arrangement allowed an additional weak coordination interaction (Zn1-O8, *vide supra*), which generated a trigonal bipyramidal geometry with severe distortion. The differences in incipient local coordination motifs should have underlined the significant differences in the extended coordination and hydrogen-bonding networks. However, with our present state of knowledge, it is formidably difficult to rationalize how the extended networks evolve from the local motifs.

### 3.2. Magnetic Properties

**Figure 4:**
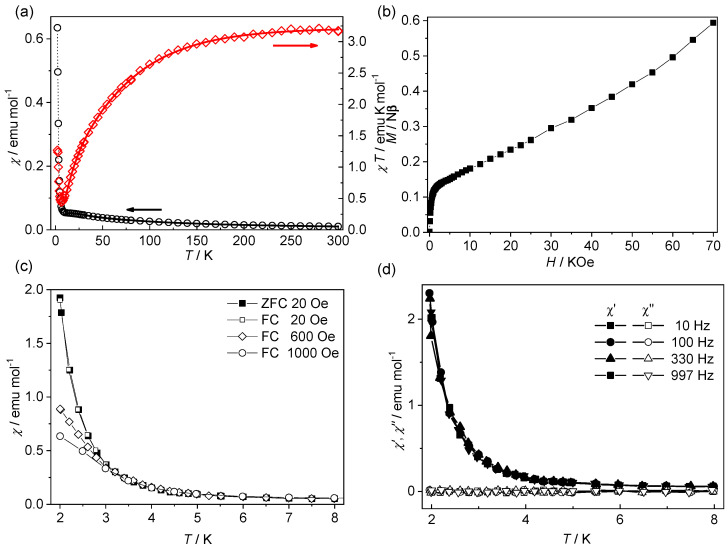
(**a**) Temperature dependence of χ and χT of **2** under 1 kOe. The black and red solid lines both represent the best fit to Equation (1) in the text. (**b**) Isothermal magnetization of **2** at 2 K. (**c**) FC and ZFC susceptibility of **2** under different field. (**d**) *χ*′(*T*) and *χ*″(*T*) plots for **2** at frequencies 10–1000 Hz with *H*_dc_ = 0 and H_ac_ = 3.5 Oe.

The molar magnetic susceptibility (*χ*) of compound **2** over the 2–300 K temperature range is displayed as *χ* and *χT* versus *T* plots in Figure 4a. The *χT* value of 3.18 emu mol^−1^ K at 300 K was obviously higher than the spin-only value (1.88 cm^3^ K mol^−1^) for *S* = 3/2 owing to the ground-state orbital degeneracy of the high-spin octahedral Co(II). The *χT* product decreased more and more rapidly as the temperature was lowered, reached a sharp minimum of 0.44 emu mol^−1^ K at 6.0 K, and rose rapidly upon further cooling. Consistently, the *χ* value witnessed an abrupt increase below 6.0 K. Fitting the data above 100 K to the Curie–Weiss law yielded a Weiss constant of *θ* = −29.7 K and a Curie constant of *C* = 3.53 cm^3^ mol^−1^ K (Figure S2).

The magnetic behaviors of Co(II) complexes are often complicated by the coexistence and interplay of the single-ion effects arising from the first-order orbital degeneracy and the interionic effects associated with magnetic interactions. Both the spin-orbital coupling of individual Co(II) ions and antiferromagnetic interactions between Co(II) ions can result in a negative Weiss constant and decreases in *χT* upon cooling. Considering that the Co(II) ions in the structure connected by the organic ligand or located in different coordination layers are far separated, effective antiferromagnetic interactions should arise from the superexchange mediated by the single-water bridges in the [Co(H_2_O)]_∞_ chain (Co···Co = 3.960(1) Å). For the quantitative interpretation of the thermal magnetic behavior, we adopt an effective-spin approach taking into account both single-ion effects and interionic magnetic exchange [53]. The Co(II) ion was assumed to have an effective spin *S*_eff_ = 1/2, corresponding to the Kramers doublet arising from spin-orbital coupling. The molar magnetic susceptibility is expressed as Equation (1), which was modified from an empirical equation for the antiferromagnetically coupled uniform chain of *S* = 1/2 spins [54] by replacing the usual *g* factor with a fictitious temperature-dependent Landé factor, *G*(*T*, *J*).

(1) $$\chi =\frac{N\beta^{2}[G(T,J){]}^{2}}{kT}\frac{0.25+0.14995x+0.30094x^{2}}{1+1.9862x+0.68854x^{2}+6.0626x^{3}}\;$$

Here, $x=25\left|J\right|/(18kT)$.

Here, *G*(*T*, *J*) is a function containing the variable parameters *λ* (spin-orbital coupling parameter), *α* (orbital reduction factor), *∆* (axial ligand-field distortion factor), and *J* (magnetic exchange parameter based on spin Hamiltonian ***H*** = −*J*Σ***S****_i_**S**_i+_*_1_). Fitting the data of **2** above 10 K to Equation (1) yielded *J* = −5.2(4) cm^−1^, *λ* = −101(5) cm^−1^, *α* = 1.45(7), and *∆* = 97(5) cm^−1^. The values of the single-ion *λ*, *α,* and *∆* parameters lay in the usual ranges for six-coordinated high-spin Co(II) complexes [25]. The negative *J* value confirmed antiferromagnetic superexchange through the single-water bridge.

The magnetic behaviors of **2** below 6.0 K indicated that the antiferromagnetic coupling along the chain did not fully cancel out the magnetic moments. There must have been a mechanism to cause the ferromagnetic-like rapid rise in *χT*. While the uniform chain structure with equivalent Co(II) centers precluded the possibility of ferrimagnetism, the behaviors could be well understood by considering a spin-canted antiferromagnetic chain. There are two possible mechanisms of spin canting [55]. One is the antisymmetric Dzyaloshinsky–Moriya (DM) interaction between adjacent spins with *g*-factor anisotropy, which favors a perpendicular arrangement of the spins and thus causes the (anti)ferromagnetically aligned spins to incline. The other is the relative inclination between the anisotropy axes of adjacent spins, which prevents the strict (anti)parallel alignment between the spins. Both mechanisms require the lack of inversion symmetry between the spin carriers. The chain in 2 met the requirement because the single-water bridge dictated an obvious canting between neighboring Co(II) octahedrons (Figure 2b). In addition, the ground-state orbital degeneracy imparted large magnetic anisotropy to Co(II). Therefore, both mechanisms of spin canting were possible for **2**. Spin canting along the antiferromagnetic chain manifests itself at low temperatures and generates noncancelled net moments, which in turn leads to ferromagnetic-like behaviors.

The spin-canting structure was supported by isothermal magnetization measurements at 2 K (Figure 4b). The magnetization showed a rapid rise upon increasing the field from 0 to 2 kOe, but it increased slowly and quasi-linearly above 2 kOe. The value (0.59 Nμ_B_) at the high field of 7 T was much lower than the saturation values generally observed for magnetically isolated octahedral Co(II) (generally 2.1–2.5 Nβ). The behavior in the high-field region confirmed the antiferromagnetic nature of the interactions through the water bridges. However, the rapid rise of magnetization in the low field region was atypical of antiferromagnetic systems and confirmed the spin-canting structure of the chain. No hysteresis was observed when the field was cycled.

Field-cooled (FC) magnetic measurements at different fields revealed that the susceptibility of **2** increased with a decreasing field at very low temperatures (Figure 4c). The field dependence was also consistent with the spin-canting structure of the antiferromagnetic chain. There was no appreciable divergence between the FC and ZFC (zero-field-cooled) data, suggesting the lack of long-range magnetic ordering between the spin-canted chains above 2 K. This was confirmed by the measurements below 8 K under an alternating-current (ac) field (Figure 4d). The ac susceptibilities showed frequency-independent in-phase signals and no out-of-phase signals.

**Figure 5:**
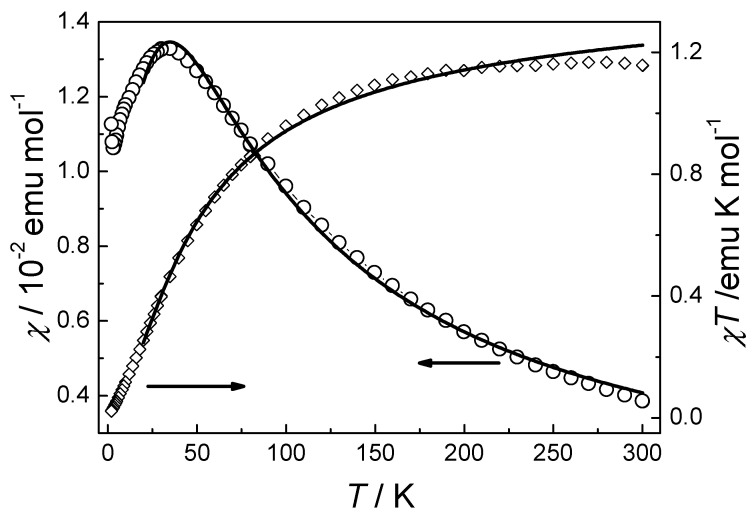
Temperature dependence of *χ* and *χT* for **3** under 1 kOe. Both solid lines represent the best fit to Equation (2) in the text.

Compound **3** exhibited a *χT* value of 1.16 emu K mol^−1^ at room temperature, consistent with a *S* = 1 system with *g* > 2. While the *χT* product increased monotonically with a decreasing temperature, the *χ* values showed a maximum at 30 K (Figure 5). These were typical of antiferromagnetic chains. The minor increase of *χ* below 4 K could have been owing to a slight number of paramagnetic impurities. **3** did not show an indication of spin canting for the lack of strong magnetic anisotropy for the Ni(II) ion. Fitting the data above 130 K to the Curie–Weiss law led to *θ* = −20.1 K with *C* = 1.25 cm^3^ mol^−1^ K (Figure S3). The magnetic exchange through the water bridge was evaluated by using Equation (2), which is based on the spin Hamiltonian ***H*** = −*J*Σ***S****_i_**S**_i+_*_1_ and valid for antiferromagnetic uniform chains with *S* = 1 [54]. The best fit of the susceptibility data to the equation yielded *J* = −18.4(1) cm^−1^ and *g* = 2.32(1).

(2) $$\chi =\frac{N\beta^{2}g^{2}}{kT}\frac{2+0.019\alpha +0.777\alpha^{2}}{3+4.346\alpha +3.232\alpha^{2}+5.834\alpha^{3}}\;$$

Here, α=|J|/kT.

The exchange parameter for Ni(II) in **3** was obviously larger in magnitude than that for Co(II) in **2**. This could have been related to the different electronic configurations, Ni(II)-*t*_2g_*^6^e*_g_^2^ and Co(II)-*t*_2g_*^5^e*_g_^2^. The parameter of overall exchange between two metal ions of the same kind is expressed as Equation (3) [54], where *n* is the number of unpaired electrons (or magnetic orbitals) for one metal ion, and the *J*_μν_ variables are the contributions from the *n^2^* exchange pathways, each involving a pair of magnetic orbitals (μ and ν):

(3) $$J=(\sum_{\mu ,\;\nu }^{n}J_{\mu \nu })/n^{2}$$

Assuming the axis defined by the single-water bridges to be the local *z*-axis for each metal ion in **2** and **3**, the two *e*_g_-type magnetic orbitals were *d*_z2_ and *d*_x2−y2_ for both Co(II) and Ni(II). The additional *t*_2g_-type magnetic orbital for Co(II) was *d*_xy_ owing to the Jahn–Teller elongation along the *z*-axis (*vide supra*). Thereby, Equation (3) becomes the following:

(4) $$J_{Ni}=(J_{d_{z2}d_{z2}}+J_{d_{x2-y2}d_{x2-y2}}+2J_{d_{z2}d_{x2-y2}})/4$$

(5) $$J_{Co}=(J_{d_{z2}d_{z2}}+J_{d_{x2-y2}d_{x2-y2}}+J_{d_{xy}d_{xy}}+2J_{d_{z2}d_{x2-y2}}+2J_{d_{z2}d_{xy}}+2J_{d_{xy}d_{x2-y2}})/9$$

The *d*_z2_ − *d*_z2_ pathway dominated the superexchange because *d*_z2_ pointed toward the water bridge and allowed a strong delocalization of spin density over the bridge. The other pathways should have made weak contributions because at least one orbital for each pathway was located in the *xy*-plane (*d*_x2−y2_ or *d*_xy_) to the disadvantage of delocalization toward the bridge. Neglecting the weak contributions and assuming *J_d_*_z2*d*z2_ (Co) ≈ *J_d_*_z2*d*z2_ (Ni) led to 4*J*_Ni_ ≈ 9*J*_Co_. This relation was qualitatively consistent with the relative magnitude of *J*_Ni_ and *J*_Co_ but underestimated the difference. Experimentally, we had 4|*J*_Ni_| (73.6 cm^−1^) > 9|*J*_Co_| (46.8 cm^−1^). The larger difference could have arisen from the following effects. (i) Owing to the larger ionic radius of Co(II) (0.74 Å) than Ni(II) (0.69 Å) and the Jahn–Teller effect of Co(II), the Co-O_water_ distance was longer than Ni-O_wate_ and led to a smaller |*J_d_*_z2*d*z2_| for Co(II). (ii) *J*_Co_ contained six pathways (the last three terms in Equation (5)) that involved orthogonal magnetic orbitals and thereby led to ferromagnetic exchange [54] whereas *J*_Ni_ had only two ferromagnetic pathways (the last term in Equation (4)). The ferromagnetic contributions reduced the magnitude of the overall antiferromagnetic interaction, and the effect was more significant for Co(II) for the larger number of ferromagnetic contributions.

There had been rare examples of Co(II) and Ni(II) compounds with single-water bridges prior to this work, among which two Co(II) and one Ni(II) compounds had been magnetically investigated [43,44]. The M-O-M bridging angles and the M···O distances fell in the ranges of 130–139° and 2.21–2.24 Å, respectively, comparable to those observed in **2** (131.8° and 2.17 Å). All these compounds showed antiferromagnetic exchange through the water bridge, which was understandable considering the rather large bridging angles on the basis of the magnetostructural correlations obtained for the hydroxo and phenoxo bridges [23,43,44,56]. However, the reported exchange parameters (−2.8 and −23.1 cm^−1^ for Co(II) and −25.9 cm^−1^ for Ni(II)) showed wide discrepancies, which could have been largely because the magnetic models for data fitting were inconsistent and adopted different approximations. In particular, without including the effects of orbital degeneracy in the magnetic analysis, the value of −23.1 cm^−1^ for Co(II) should have been overestimated [44]. More experimental examples, as well as theoretical calculations, are demanded to gain reliable magnetostructural correlations for the bridge.

Finally, the single-water bridge was compared with the single-carboxylate bridge in terms of their efficiency in magnetic exchange. Carboxylate can take different bridging modes between metal ions, which strongly affect magnetic exchange. The single-carboxylate bridge as a stand-alone group connecting two metal ions is much less common than multiple carboxylate bridges or mixed systems containing other bridges. The single-carboxylate bridge taking the *anti-syn* or *anti-anti* mode has been found in Co(II) and Ni(II) coordination polymers. The magnetic exchanges mediated by the bridge varies from weakly antiferromagnetic to weakly ferromagnetic. The *J* values lies in the ranges of −2–+0.2 cm^−1^ for Co(II) and −4–+1 cm^−1^ for Ni(II) [57,58,59,60,61,62,63]. Obviously, the single-water bridge mediates stronger magnetic exchange than the single-carboxylate bridge in the *anti-syn* or *anti-anti* mode.

## 4. Conclusions

Three coordination polymers were synthesized from a nitro-tagged biphenyldicarboxylate ligand. Structural analysis demonstrated that water molecules played essentially important structural roles. In **1**, terminally coordinated water molecules afforded hydrogen-bonded ladders to connect the metal-dicarboxylate chains. For isomorphous **2** and **3**, water molecules served as single bridges to form [M(H_2_O)]_∞_ chains, which intersected with carboxylate chains to yield 2D coordination networks. The water bridge in **2** and **3** also played a functional role. It propagated antiferromagnetic interactions along the Co(II) or Ni(II) chain and caused a spin-canted magnetic structure for the Co(II) chain.

## Figures and Tables

**Table 1:** Crystal data and structure refinement for compounds **1** and **2**.

Compound	1	2
Formula	C_17_H_16_ZnN_2_O_8_	C_17_H_20_CoN_2_O_10_
CCDC	2406943	2406942
Formula Weight	441.69	471.28
Temperature, K	293	293
Crystal system	Monoclinic	Monoclinic
space group	*P*2_1_*/c*	*P*2_1_*/c*
*a*, Å	7.238(4)	14.947(2)
*b*, Å	27.774(13)	17.226(2)
*c*, Å	9.562(5)	7.9198(11)
*α*, deg	90	90
*β*, deg	108.233(6)	95.989(2)
*γ*, deg	90	90
*V,* Å^3^	1825.9(15)	2028.1(5)
Z	4	4
*D*_c_, g cm^−3^	1.607	1.543
*μ*, mm^−1^	1.394	0.904
Reflns collected	8144	9013
Unique reflns/ *R*_int_	3591/0.0331	3949/0.0484
*R*_1_ [*I* > 2*σ*(*I*)]	0.0383	0.0725
*wR*_2_ (all data)	0.0941	0.1870
GOF	1.082	1.118

## Data Availability

The original contributions presented in this study are included in the article/supplementary material. Further inquiries can be directed to the corresponding authors.

## Supplementary Materials

The following supporting information can be downloaded at: https://www.mdpi.com/article/10.3390/ma18051089/s1. Table S1: Hydrogen bond lengths (Å) and angles (°) for compounds **1** and **2**; Figure S1: FT-IR of compounds **1**, **2** and **3**; Figure S2: Temperature dependence of *χ*^−1^ for **2** under 1 kOe; Figure S3: Temperature dependence of *χ*^−1^ for **3** under 1 kOe. X-ray data for compound **1** (CCDC 2406943) and compound **2** (CCDC 2406942).
