# Supplementary material for: Roles of Water Molecules in the Structures and Magnetic Properties of Coordination Polymers with a Dicarboxylate Ligand
Source: Materials (Basel). 2025 Feb 28;18(5):1089. doi: 10.3390/ma18051089 (PMC11901891; doi:10.3390/ma18051089)
Supplement: Supplementary file 1 [file materials-18-01089-s001.zip › materials-3497775-supplementary.pdf]

Roles of water molecules in the structures and magnetic properties of coordination polymers with a dicarboxylate ligand

Dehui Zong, En-Qing Gao* and Dawei Zhang*

State Key Laboratory of Petroleum Molecular & Process Engineering, Shanghai Key Laboratory of Green Chemistry and Chemical Processes, School of Chemistry and Molecular Engineering, East China Normal University, Shanghai 200062, China

Table S1. Hydrogen bond lengths (Å) and angles (°) for compound **1** and **2**

D-H...□	d(D-H)	d(H...□)	d(D...□)	<(DH□)
Compound **1**				
O2-HW2□...O4#1	0.83(4)	1.82(5)	2.637(4)	172(4)
O2-HW2B...O8#2	0.76(4)	1.91(4)	2.659(4)	169(4)
Compound **2**				
O4-HW4□...O6	0.83(2)	1.94(3)	2.718(7)	156(6)
O4-HW4B...O1#3	0.84(2)	2.23(9)	2.926(6)	140(12)
O4-HW4B...O5#3	0.84(2)	2.43(13)	2.997(6)	125(13)
O3-HW3□...O9#2	0.846(19)	1.85(2)	2.658(5)	159(5)
O3-HW3B...O2#4	0.851(19)	1.745(19)	2.584(4)	169(5)
O6-HW6□...O2#4	0.86(2)	2.00(7)	2.762(7)	146(11)
O6-HW6B...O9#5	0.87(2)	2.11(2)	2.876(7)	146(4)

Symmetry codes: #1, x+1, y, z, #2, x+1, 1/2-y, 1/2+z, #3, x, 1/2-y, 1/2+z, #4, x, 1/2-y, z-1/2, #5 -x, y-1/2, -z-1/2

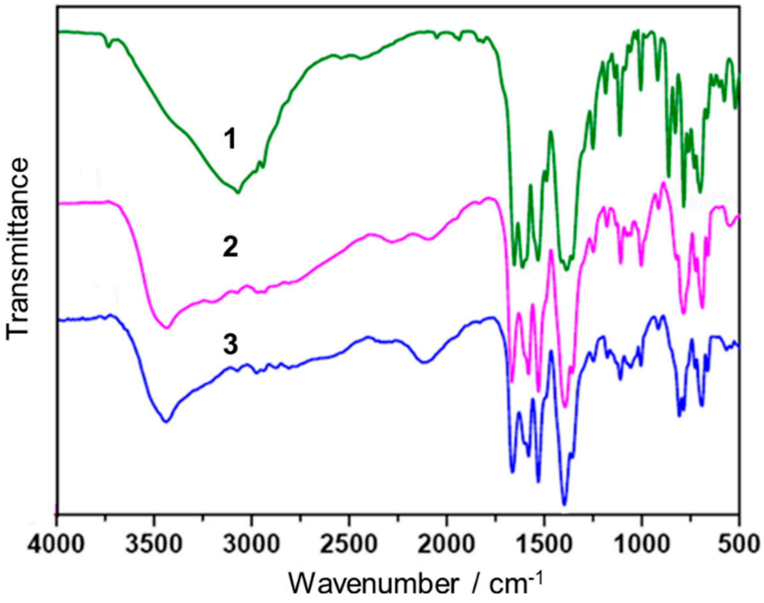

Figure S1. FT-IR of compounds **1**, **2** and **3**.

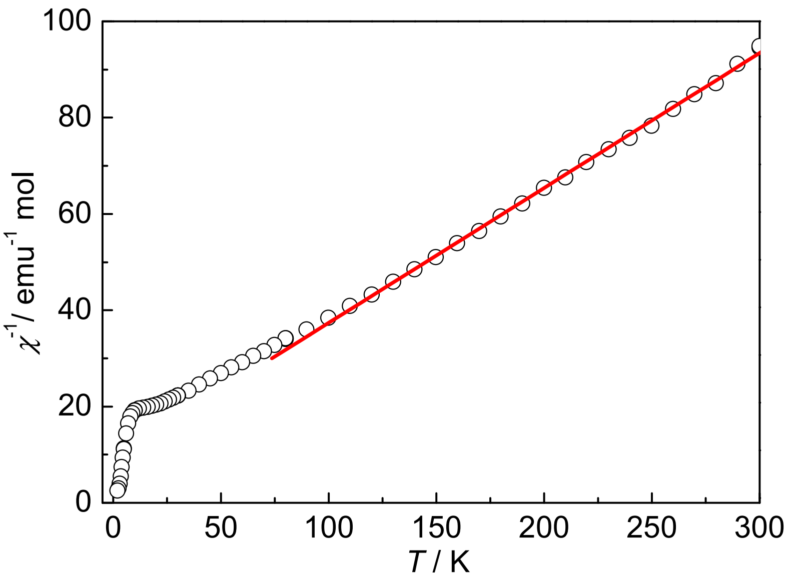

Figure S2. Temperature dependence of χ^{-1} for **2** under 1 kOe.

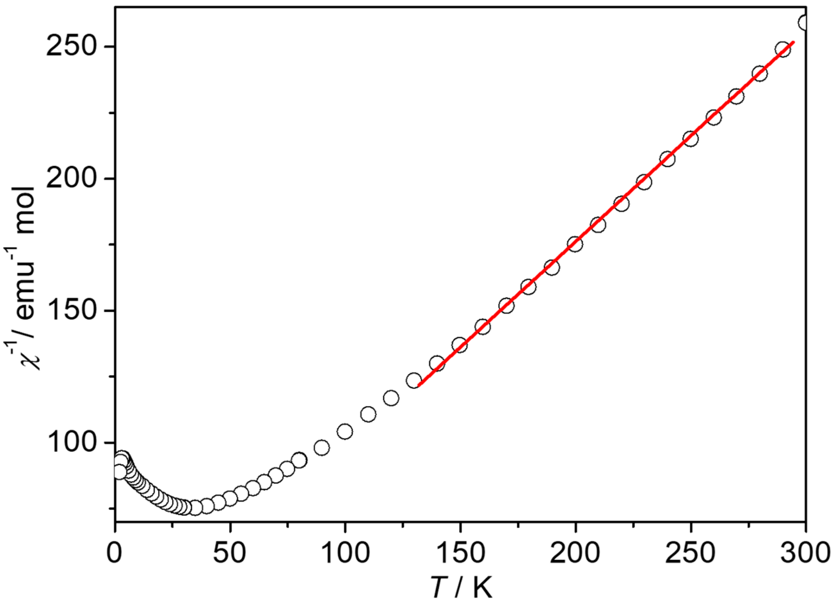

Figure S3. Temperature dependence of χ^{-1} for **3** under 1 kOe.
